# *In Silico* Prediction and *In Vitro* Characterization of Multifunctional Human RNase3

**DOI:** 10.1155/2013/170398

**Published:** 2013-01-17

**Authors:** Pei-Chun Lien, Ping-Hsueh Kuo, Chien-Jung Chen, Hsiu-Hui Chang, Shun-lung Fang, Wei-Shuo Wu, Yiu-Kay Lai, Tun-Wen Pai, Margaret Dah-Tsyr Chang

**Affiliations:** ^1^Institute of Molecular and Cellular Biology, National Tsing Hua University, No. 101, Section 2, Kuang Fu Road, Hsinchu 30013, Taiwan; ^2^Institute of Biotechnology, National Tsing Hua University, No. 101, Section 2, Kuang Fu Road, Hsinchu 30013, Taiwan; ^3^Department of Computer Science and Engineering, National Taiwan Ocean University, 2 Pei Ning Road, Keelung 20224, Taiwan; ^4^Department of Medical Science, National Tsing Hua University, No. 101, Section 2, Kuang Fu Road, Hsinchu 30013, Taiwan

## Abstract

Human ribonucleases A (hRNaseA) superfamily consists of thirteen members with high-structure similarities but exhibits divergent physiological functions other than RNase activity. Evolution of hRNaseA superfamily has gained novel functions which may be preserved in a unique region or domain to account for additional molecular interactions. hRNase3 has multiple functions including ribonucleolytic, heparan sulfate (HS) binding, cellular binding, endocytic, lipid destabilization, cytotoxic, and antimicrobial activities. In this study, three putative multifunctional regions, ^34^RWRCK^38^ (HBR1), ^75^RSRFR^79^ (HBR2), and ^101^RPGRR^105^ (HBR3), of hRNase3 have been identified employing *in silico* sequence analysis and validated employing *in vitro* activity assays. A heparin binding peptide containing HBR1 is characterized to act as a key element associated with HS binding, cellular binding, and lipid binding activities. In this study, we provide novel insights to identify functional regions of hRNase3 that may have implications for all hRNaseA superfamily members.

## 1. Introduction

Human ribonuclease A (hRNaseA) family members are encoded by unique genes located on human chromosome 14 [1]. The hRNaseA family is vertebrate cationic protein sharing conserved tertiary structure and specific enzymatic sites for RNase activity. It is in general considered to comprise eight members: RNase1 (pancreatic RNase), RNase2 (eosinophil derived neurotoxin/EDN), RNase3 (eosinophil cationic protein/ECP), RNase4, RNase5 (angiogenin), RNase6, RNase7 (skin-derived RNase), and RNase8 (divergent paralog of RNase7) [2]. Analysis of human genome sequence has revealed the existence of five additional RNases named as RNases9–13, although they appear to lose enzymatic activity [3]. All hRNaseA family members encode relatively small polypeptides of 14 to 16 kDa containing signal peptides of 20 to 28 amino acids for protein secretion. Mature hRNaseA members contain 6 to 8 cysteine residues that are crucial to hold the overall tertiary structure [4]. They possess an invariant catalytic triad including two histidines (one near the *N* terminus, and the other near the *C* terminus) and one lysine located within a conserved signature motif (CKXXNTF) [5]. These RNaseAs are catalytically active to various degrees against standard polymeric RNA substrates [6]. Interestingly, their host defense functions including cytotoxic [7, 8], helminthotoxic [9, 10], antibacterial [11, 12], and antiviral [5, 13] activities have also been reported. However, the mechanisms of noncatalytic functions of some hRNaseA members, especially the ones with low RNase activities, are poorly understood. 

hRNase3 is found within the secondary granules of eosinophils and serves as a clinical asthma marker [14]. It is a multiple functional protein as the *N*-terminal domain^1–45^ possesses antipathogenic activities such as antibacterial, antihelminthic, and antiviral competencies [15–17]. In terms of key amino acids involving specific functions, Trp^35^ of hRNase3 interacts with cell membrane to form transmembrane pores in an artificial lipid bilayer, suggesting that lipid destruction is a crucial step in bactericidal activity [18]. In addition, Arg^1^, Trp^10^, Gln^14^, Lys^38^, and Gln^40^ located in antipathogenic domain are identified to bind to lipopolysaccharide (LPS) and peptidoglycan with high affinity, which may also be important for its bactericidal activity [19, 20]. Moreover, hRNase3 possesses cytotoxic activity against various mammalian cell lines including those derived from blood and epidermis [17], and Arg^97^ is the key residue for its cytotoxicity [21]. It is also highly associated with host inflammatory response and thus involved in tumor microenvironment to exercise its antitumor response [22–24]. Interestingly, hRNase3 reduces the infectivity of human respiratory syncytial virus in an RNase activity-dependent manner [25]. Treatment of bronchial epithelial cells with hRNase3 induces production of tumor necrosis factor alpha (TNF-*α*) and triggers apoptosis via a caspase-8-dependent pathway [26]. Furthermore, hRNase3 is reported to bind to a class of cell surface receptors termed as heparan sulfate proteoglycans [27] and thereby internalizes target cells through macropinocytosis [28]. Taken together, basic and aromatic residues, especially Arg and Trp, are considered to play important roles in enzymatic and other biological functions of hRNase3.

In this study, in combination with *in silico* analyses employing Reinforced Merging for Unique Segments (ReMUS) system, we have identified three heparin binding regions (HBRs) in hRNase3. We focused on their roles in heparin and cellular binding and endocytic and cytotoxic activities employing *in vitro* functional analyses. Our results showed that HBR1 (^34^RWRCK^38^) is crucial for enzymatic RNase function and serves as a major heparin binding site for endocytosis, HBR2 (^73^RSRFR^77^) contributes toward cell binding and endocytic activities, and HBR3 (^101^RPGRR^105^) plays a critical role in cytotoxicity. In addition, a noncytotoxic HBR1-derived peptide was characterized to bind to negatively charged molecules including glycosaminoglycans (GAGs) and lipids on cell surface. In summary, we have identified multifunctional regions of hRNase3, which may provide novel insights to implicate for all hRNaseA superfamily members.

## 2. Materials and Methods

### 2.1. *In Silico* Analysis

 Unique peptides of query proteins, 13 hRNaseA family members, were identified employing Reinforced Merging for Unique Segments ReMUS system (ReMUS) (http://140.121.196.30/remus.asp) [42]. The system adopted a bottom-up strategy to extract unique patterns in each sequence at different unique levels. A fundamental unique peptide segment with previously defined pattern length, named as primary pattern was extracted at the first step. The rule of thumb for primary pattern lengths is that a shorter length setting for similar protein sequences and a longer length for dissimilar ones. The length of primary pattern in this study is set as 3 residues for hRNaseA protein family. After that Boyer Moore algorithm was performed to efficiently retrieve all primary patterns among all sequences. Each verified fundamental unique peptide segment was analyzed based on its frequencies of appearance, and its representation level of uniqueness was calculated for the merging processes in the next module. The last merging algorithm concatenated these extracted unique peptide segments through a bottom-up approach only if the primary unique peptide segments were overlapped within a sequence. The merged segments were guaranteed with unique features compared to all other protein sequences in the query dataset.

Clustal W2 (http://www.ebi.ac.uk/Tools/msa/clustalw2/) was used to align protein sequences of 13 hRNaseA family members on the basis of automatically progressive alignment mode. Protein sequences were retrieved from UniProtKB (http://www.uniprot.org/). To perform multiple-sequence alignment, gap open and extend penalties were set to 10 and 0.2, respectively. For secondary structure analysis in corresponding HBRs of hRNase1 to hRNase8, tertiary structures of hRNase1, 2, 3, 4, 5, and 7 were collected from protein data bank (PDB, http://www.rcsb.org/pdb/home/home.do), and those of hRNase6 and hRNase8 were simulated by Protein Structure Prediction Server (PS)^2^ (http://ps2.life.nctu.edu.tw/) using hRNase7 as a template. In addition, National Center for Biotechnology Information (NCBI) Blast (http://blast.ncbi.nlm.nih.gov/Blast.cgi) was employed to compare sequence correspondent to HBP_RNase3(32–41)_ among nonhuman primate hRNase2s and hRNase3s, as well as human RNaseA superfamily members.

### 2.2. Cell Line Strains

Beas-2B (ATCC number: CRL-9609) was a human lung bronchial epithelial cell line infected with an adenovirus 12-SV40 virus hybrid (Ad12SV40). Beas-2B cells were cultured in RPMI 1640 medium (Gibco, Invitrogen, USA) supplemented with 56°C heat-inactivated 10% (v/v) fetal bovine serum (FBS) (Gibco, Invitrogen, USA) and 1% (v/v) Glutamine-Penicillin-Streptomycin (Biosera). Wild-type Chinese Hamster Ovary- (CHO-) K1 and mutant cell lines CHO-pgsA-745 (lacking of all GAGs) and CHO-pgsD-677 (lacking of HS) were cultured in Vitacel Ham's F12 K medium (Sigma-Aldrich) supplemented with 10% FBS. Beas-2B and pgs D-677 cell lines were purchased from ATCC. CHO-K1 and CHO-pgsA745 cell lines were kindly provided by Dr. W.-G. Wu and Dr. C.-L. Yang, respectively (Department of Life Science, National Tsing Hua University, Taiwan). The cells were maintained at 37°C in a humidified atmosphere of 5% CO_2_. 

### 2.3. Recombinant Protein Purification

Recombinant wild-type hRNase3 with a *C*-terminal His_6_ tag was expressed in *Escherichia coli*  BL21(DE3) Codon Plus (Novagen), purified by affinity column chromatography, and refolded as previously described [27, 43]. The plasmids containing insert encoding mutant HBR1, HBR2, and HBR3 hRNase3 were generated by QuikChange site-directed mutagenesis with primer sets mtr1 forward: 5′-TATGCAGCGGCTTGCGCAAACCAAAAT-3′ and mtr1 reverse 5′-TTTGCGCAAGCCGCTGCATAATTGTTA-3′; mtr2 forward 5′-AGGCACGGCGGCGGCGGCGGCATGACAATTGTTGAG-3′ and mtr2 reverse 5′-TGTCATGCCGCCGCCGCCGCCGTGCCTTTACTCCAC-3′; mtr3 forward 5′-ATAGAAGGCGGCGGCGGCGGCGTCTGCATACCTGCA-3′ and mtr3 reverse 5′-GCAGACGCCGCCGCCGCCGCCTTCTATGTAGTTGCA-3′. For each preparation, 10 mL of overnight culture was subcultured into 1 L TB containing 100 *μ*g/mL ampicillin, and grown at 37°C for 6 h. Isopropyl-*β*-D-thiogalactopyranoside (IPTG) was added to a final concentration of 0.5 mM. Wild-type and mutant hRNase3 were collected from inclusion bodies that were refolded by dialysis in refolding buffer (20 mM Tris, 0.5 M arginine, 0.2 mM GSSG, 2 mM EDTA, 10% glycerol, pH 8.5) at 4°C gently, concentrated by Amicon Ultra-15 (Millipore), and stored in phosphate-buffered saline (PBS). 

### 2.4. Fluorescence-Assisted Carbohydrate Electrophoresis (FACE)

Carbohydrates were labeled with 2-aminoacridone (AMAC) according to previous study [44]. The AMAC-labeled carbohydrate and peptide were mixed and incubated at 25°C for 15 min. The complex was then loaded onto 1% agarose gels and electrophoresed in the buffer containing 40 mM Tris-acetic acid, 1 mM EDTA, pH 8.0 for 20 to 30 min. This experiment was performed in dark or under red light to prevent from light exposure. The AMAC labeled probe was observed under UV light (424 nm) and scanned by transilluminator.

### 2.5. RNase Activity Assay

RNase activity assay of recombinant wild-type and mutant hRNase3 were performed using yeast tRNA (Invitrogen), and bovine RNaseA (USB) was typically RNase and used as positive control. Three hundred microliters of 100 mM sodium phosphate (NaPO_4_) buffer, pH 7.4, and 500 *μ*L diethylpyrocarbonate- (DEPC-) ddH_2_O were mixed including 50 *μ*L of 0.05 *μ*M RNaseA and 5 *μ*M of wild-type and mutant hRNase3, separately. Ten microliters of 5 mg/mL yeast tRNA was added and incubated at 37°C for 0, 5, 10, and 15 min, respectively. Ice-cold 500 *μ*L stop solution (1 : 1 (v/v) 40 mM lanthanum nitrate and 6% perchloric acid) was added and mixed for 10 min to stop reaction. Entire yeast tRNA was suspended by centrifugation at 16,100 ×g at 25°C for 5 min. One hundred microliters of supernatant in each tube was placed on to a 96-well plate. The amount of soluble tRNA in supernatant was determined by UV absorbance at 260 nm.

### 2.6. MTT Assay

Beas-2B cells (5 × 10^3^cells/well) were plated in each well of a 96-well plate and allowed to incubate at 37°C for 24 h. Beas-2B cells were incubated with 0, 5, 10, 20, 40, 60, 80, and 100 *μ*M HBP_RNase3(32–41)_ and were incubated at 37°C for 24 h. 3-(4,5-Dimethylthiazol-2-yl)-2,5-diphenyltetrazolium bromide (MTT) solution (10 *μ*L of 10 mg/mL, Roche) was added to each well, and the cultures were incubated for an additional 4 h. After 24 h, the culture medium was replaced by 100 *μ*L 5 mg/mL MTT for 3 h, then MTT solution was substituted by 100 *μ*L 100% DMSO for 15 min. Finally, the absorbance of the sample was measured at 570 nm.

### 2.7. Uptake Assay and Western Blotting

Beas-2B cells were seeded into 100 mm dish at 37°C for 24 h and followed by washed with PBS and incubated with 1 *μ*M wild type or mutant hRNase3 in serum-free RPMI 1640 medium at 37°C for 1 h. The cells were washed with PBS and trypsinized for at 37°C 15–20 min to remove surface-associated protein, and cells were lysed with Pro-prep (iNtRon). After electrophoresis, proteins were transferred to PVDF membrane (Pall) using a Bio-Rad Trans-Blot semidry transfer cell. Membranes were blocked with 3% BSA in 0.15 M NaCl, 0.5% (v/v) Tween-20, and 20 mM Tris, pH 7.4 (TBST) followed by primary antibody (anti-His antibody) diluted in 1% BSA/TBST. After washing with TBST, membranes were incubated with a secondary antibody (anti-mouse antibody) diluted in 1% BSA/TBST. Bound antibody was detected using the enhanced chemiluminescence detection system (Pierce).

### 2.8. Synthetic Peptides

HBP_RNase3(32–41)_ (NYRWRCKNQNK), HBP_RNase3(71–80)_ (NNCHRSRFRV) and HBP_RNase3(96–105)_ (TYADRPGRRF) were synthesized by Genemed Synthesis Inc. (USA). All peptides were purified by analytical high-pressure liquid chromatography to a purity exceeding 90%. The identity of peptides was confirmed by matrix-assisted laser desorption ionization time of flight mass spectrometry.

### 2.9. Cell-Based Enzyme-Linked Immunosorbent Assay (cELISA)

Cells (2 × 10^4^ cells/well) were seeded in a 96-well black plate and incubated with 5% CO_2_ at 37°C for 24 h. The fluorescein isothiocyanate- (FITC-) labeled HBP_RNase3(32–41)_,  HBP_RNase3(71–80)_, and HBP_RNase3(97–106)_ were diluted to 0, 1, 5, and 10 *μ*M in medium for 1 h. After wash with 100 *μ*L PBS, the plate was fixed by 2% (w/v) paraformaldehyde (PFA) in PBS for 15 min, and 100 *μ*L 50 mM ammonium chloride in PBS was added to quench fluorescence. The plate was washed with 100 *μ*L PBS, and 2% (w/v) BSA in PBS was added to block at room temperature for 1.5 h. The signals were measured using 488 nm laser with standard 530 nm ± 30 nm bandpass emission filter (Omega Optical, Brattleboro, VT, USA). In each set of the assays, analyses were carried out in duplicate or triplicate.

### 2.10. Lipid Overlay Blots

SphingoStrips (Invitrongen catalog no. S23753) and PIP Strips (Invitrongen catalog no. P23751) membranes were blocked at 37°C for 1 h with Tris-buffered Saline/0.05% Tween-20 (TBST) containing 3% (w/v) fatty acid free BSA. Membranes were incubated with 1 *μ*g/mL hRNase3 or 0.5 *μ*g/mL FITC-HBP_RNase3(32–41)_ at 37°C for 2 h separately, the membranes were washed once using 0.1% BSA/TBST with a gentle shaking for 10 min. hRNase3 binding to lipids was probed by primary antibody (mouse anti-6His 1 : 5000) for 1 h. After wash, anti-mouse antibody conjugated with horseradish peroxidase (HRP) was used as secondary antibody (1 : 5000) in 1% BSA/TTBS. Finally, the immunoreactive bands were visualized by enhanced chemiluminescence (USA). For FITC-HBP_RNase3(32–41)_ analysis, the membrane strips were initially incubated with FITC-HBP_RNase3(32–41)_ followed by PBS wash and finally imaged using 488-nm laser with standard 530 nm ± 30 nm bandpass emission filter.

## 3. Result

### 3.1. *In Silico* Analysis of Unique Peptide Regions in hRNaseA Superfamily

**Figure 1:**
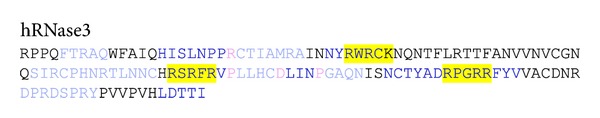
Unique peptide motifs in hRNase3 revealed from 13 hRNaseA members by Reinforced Merging for Unique Segments (ReMUS) system. Blue and light blue segments represent unique peptide motifs of hRNase3 compared to other 12 hRNaseA members. Three unique motifs ^34^RWRCK^38^, ^75^RSRFR^79^, and ^101^RPGRR^105^, representing, respectively, HBR1, HBR2, and HBR3 in hRNase3 are highlighted in yellow.

**Figure 2:**
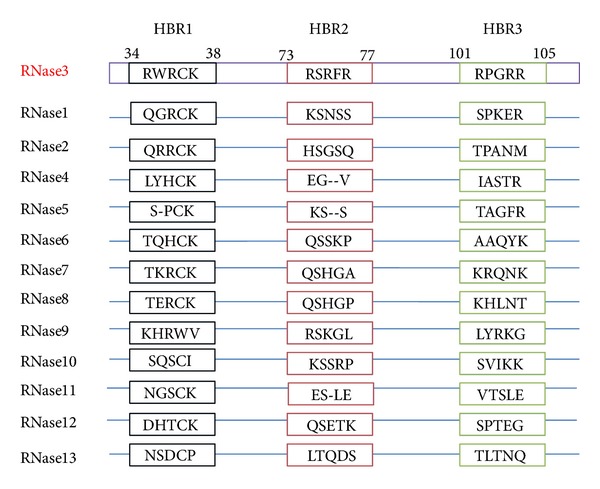
Sequences of HBR1, HBR2, and HBR3 in hRNase3 and corresponding regions of other 12 hRNaseA members. Sequences of hRNase3 and other hRNaseA superfamily members are aligned using ClustalW2 software. Putative HBR1, HBR2, and HBR3 separately located on residues 34–38, 73–77, and 101–105 are predicted from hRNase3. Residues in black, red, and green boxes indicate corresponding sequence motifs aligned with, respectively, HBR1, HBR2, and HBR3 in all hRNaseA superfamily members.

To predict unique peptide regions possibly involved in multifunctions of hRNase3, ReMUS system was employed to analyze sequences of hRNase3 and the other 12 members of hRNaseA family. Eleven unique peptide motifs including HISLNPPR, RCTIAMRA, NYRWRC, SIRCPHNRTLNNC, RSRFRVP, PLLHCD, DLINP, PGAQN, NCTYADRPGRRFYV, DPRDSPRY, and LDTTI in hRNase3 were identified as shown in blue and light blue in Figure 1. Among which three unique segments rich in positively charged amino acids were denoted as putative HBRs including ^34^RWRCK^38^ (HBR1), ^73^RSRFR^77^ (HBR2), and ^101^RPGRRR^105^ (HBR3). Since heparin binding activity of HBR1 has been previously reported [27], the presence of 3 HBRs might possibly correlate with stronger heparin binding features of hRNase3 than other hRNaseA family members. Subsequently, Clustal W2 was applied to compare primary sequence of hRNase3 with the other hRNaseAs and alignment of putative HBRs of hRNase3 with correspondent segments of the other 12 hRNaseAs.  Figure 2 revealed that HBR1 in hRNase3 was 60% identical to the corresponding segments of hRNase1, hRNase2, hRNase7, and hRNase8, but these HBRs were not conserved with any of the other hRNase family members, suggesting that these three HBRs might account for unique functions of hRNase3. Therefore, seven mutant hRNase3 constructs were generated by site-directed mutagenesis with selective alanine replacement in each HBR in order to investigate unique functions of HBR1, HBR2 and HBR3 in hRNase3 (Supplementary Figure 1 available online at http://dx.doi.org/10.1155/2013/170398).

### 3.2. RNase Activity of Wild-Type and Mutant RNase3

**Figure 3:**
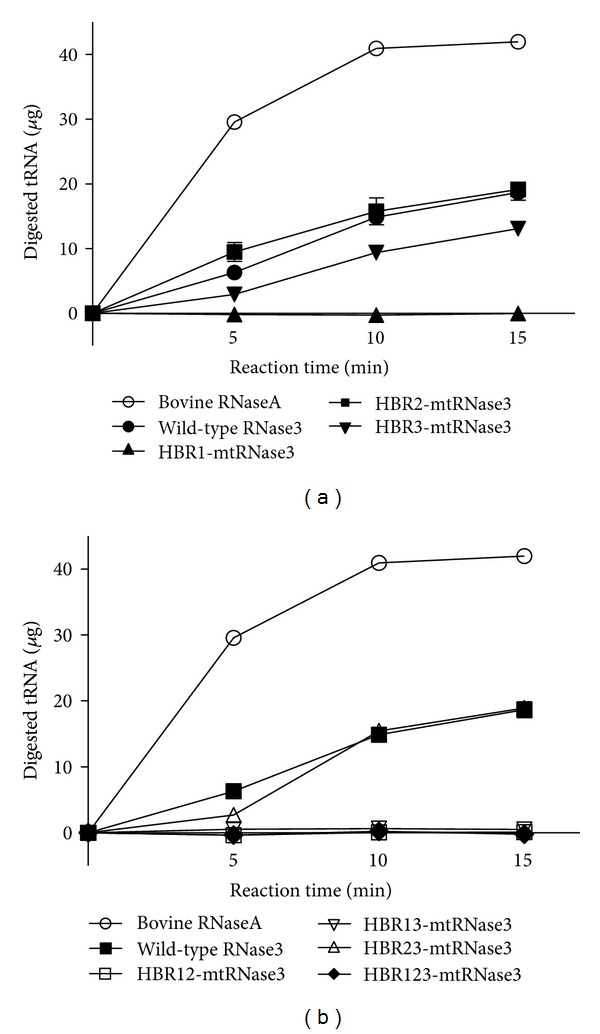
RNase activity of wild-type and mutant hRNase3. Five mg of yeast tRNA was added to 0.05 *μ*M bovine RNaseA and 5 *μ*M of (a) HBR1-mtRNase3, HBR2-mtRNase3, and HBR3-mtRNase3 and (b) HBR12-mtRNase, HBR13-mtRNase3, HBR23-mtRNase3, and HBR123-mtRNase3 separately to examine the RNase activity. The amount of digested ribonucleotides in supernatant was detected by monitoring OD_260_ and RNase activity of bovine RNaseA was set as a positive control.

After 0.05 *μ*M bovine RNaseA, 5 *μ*M wild-type or mutant hRNase3 was incubated with 50 *μ*g tRNA at 37°C for 0, 5, 10, and 15 min, the amount of digested ribonucleotides was examined by UV absorbance at 260 nm.  Figure 3(a) showed that RNase activity of HBR1-mt RNase3 was significantly reduced and that of HBR3-mt RNase3 was 15% less than wild type hRNase3. Besides, the RNase activity of HBR2-mt RNase3 was comparable to that of wild-type hRNase3. Moreover, double and triple mutation abolished hRNase activities of HBR12-, HBR13- and HBR123-mtRNase3 expect HBR23-mtRNase3 (Figure 3(b)). These results suggested that HBR1 of hRNase3 played a critical role in ribonucleolytic activity, mainly due to the presence of a catalytic residue Lys in the sequence. 

### 3.3. Endocytosis Activity of Wild-Type and Mutant hRNase3 to Beas-2B Cells

**Figure 4:**
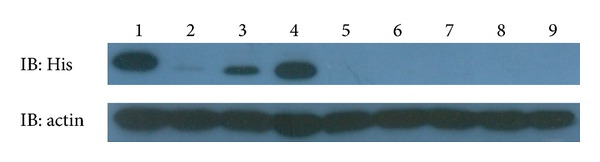
Endocytosis of wild-type and mutant hRNase3 to Beas-2B cells. Endocytosis activity of hRNase3 and its HBR mutants were assayed by western blotting employing 1 : 5000 dilution of anti-His-antibody. Actin was used to normalize the hRNase3-6His immunoblot signal. Forty micrograms of total protein in cell lysates were separated by 15% SDS-PAGE. IB: immunoblot; lane 1: wild-type hRNase3; lane 2-HBR1-mtRNase3; lane 3-HBR2-mtRNase3; lane 4-HBR3-mtRNase3; lane 5-HBR12-mtRNase3; lane 6-HBR13-mtRNase3; lane 7-HBR23-mtRNase3; lane 8-HBR123-mt RNase3; lane 9-cell only.

To determine the influence of different HBRs on endocytosis activity of hRNase3, intracellular uptake assay was performed. Beas-2B cells were treated with wild type or mutant hRNase3 at 37°C for 1 h, followed by trypsin digestion for 15 min to remove surface-bound recombinant proteins before being analyzed by western blotting. When 40 *μ*g of total cell lysates were examined with an exposure time of 1 min, only HBR2-mtRNase3 and HBR3-mtRNase3 could be detected in cytosol of Beas-2B cells (Figure 4, lanes 3 and 4). In addition, none of the double and triple HBR mutants of hRNase3 was able to enter Beas-2B cells (Figure 4, lanes 5, 6, 7, and 8). These results indicated that the importance of HBRs associated with endocytosisactivity of hRNase3 to Beas-2B cells in increasing order was HBR3, HBR2, and HBR1.

### 3.4. Cytotoxicity of Wild-Type and Mutant hRNase3 to Beas-2B Cells

**Figure 5:**
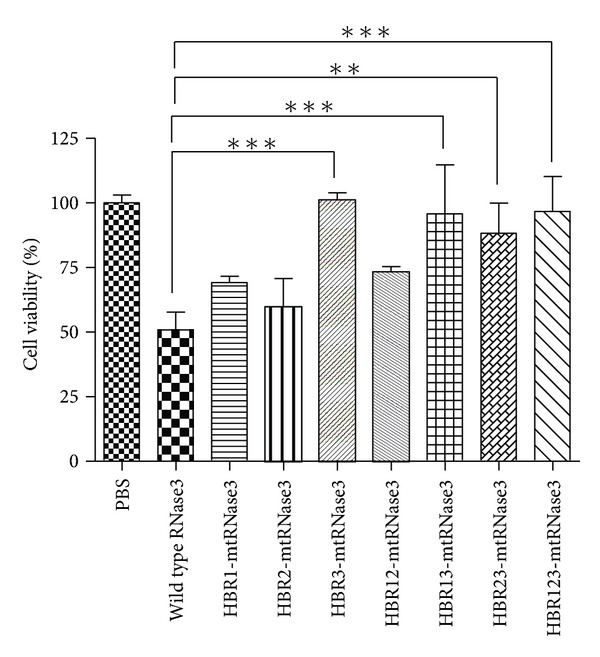
Cytotoxicity of wild-type and mutant hRNase3 to Beas-2B cells. Cytotoxicity of hRNase3 and its HBR mutants to Beas-2B cells were assessed by MTT assay. Cell viability of Beas-2B cells with PBS treatment was set as 100%. Results are presented as mean ± SD (*n* = 3). **P < 0.01, ***P < 0.005.

Beas-2B cells were incubated individually with 15 *μ*M of wild-type or mutant hRNase3 in serum-free medium at 37°C for 48 h followed by MTT assay. The cell viability of PBS treatment to Beas-2B cells was set as 100% to normalize that of different protein treatment.  Figure 5 revealed that the viability of wild-type hRNase3-treated cells decreased to 50%, while that of HBR1-, HBR2-, HBR3-, HBR12-, HBR13-, HBR23-, and HBR123-mtRNase3-treated cells increased to 69%, 60%, 101%, 73%, 96%, 88%, and 97%, respectively. The cytotoxicity of HBR3-mtRNase3, HBR13-mtRNase3, HBR23-mtRNase3, and HBR123-mtRNase3 apparently diminished as compared to that of wild-type hRNase3, suggesting that HBR3 mutation played a major role in loss of cytotoxicity of hRNase3. It should be noted that HBR3 was located on *β* sheet 6 of hRNase3, hence mutation of HBR3 to alanine stretch might possibly lead to conformational change and subsequent of functional variation. 

### 3.5. Binding Activity of Wild Type and Mutant hRNase3 to LMWH

**Figure 6:**
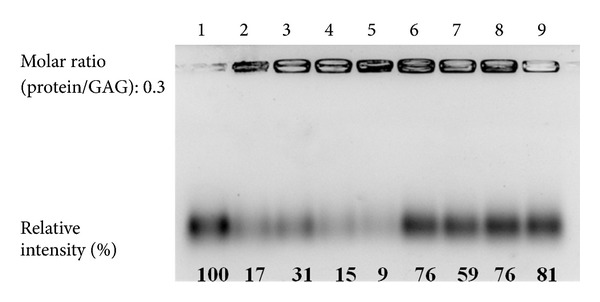
Binding activity of wild-type and mutant hRNase3 to LMWH. Heparin binding activity of hRNase3 and its HBR mutants were assessed by FACE. The value of labeled LMWH in the absence of protein was measured and set as 100%. Relative intensity of LMWH signal of each protein was normalized to LMWH only signal and was marked at the bottom; lane 1-LMWH only; Lane 2-hRNase3; lane 3-HBR1-mtRNase3; lane 4-HBR2-mtRNase3; lane 5-HBR3-mtRNase3; lane 6-HBR12-mtRNase3; lane 7-HBR13-mtRNase3; lane 8-HBR23-mtRNase3; lane 9-HBR123-mt RNase3.

The influence of wild type and mutant hRNase3 on LMWH binding activity was illustrated in Figure 6. Initially, AMAC-labeled LMWH was coincubated with wild-type or mutant hRNase3 individually at a molar ratio of protein to LMWH of 0.3. Free probe was separated by 1% gel electrophoresis. Relative intensities of free probes of wild type, HBR1-, HBR2-, HBR3-, HBR12-, HBR13-, HBR23-, and HBR123-mt RNase3 apparently decreased to, respectively, 17%, 31%, 15%, 9%, 76%, 59%, 76%, and 81%. The binding activity of HBR1-mtRNase3 to LMWH decreased 14% as compared to that of wild type hRNase3 (Figure 6, lanes 2 and 3), while HBR2- and HBR3-mt RNase3 did not show much difference in LMWH binding activity (Figure 6, lanes 4 and 5). However, the binding activity of double- and triple-mutant hRNase3 to LMWH decreased more significantly, especially when HBR1 was mutated (Figure 6, lanes 6, 7, 8, and 9). Therefore, in terms of facilitating hRNase3 binding to LMWH, the importance of three HBRs in decreasing order was HBR1, HBR2, and HBR3.

### 3.6. Binding Activity of Synthetic Peptides FITC-HBP_RNase3(32–41)_, FITC-HBP_RNase3(71–80)_, and HBP_RNase3(97–106)_ to Beas-2B Cells

**Figure 7:**
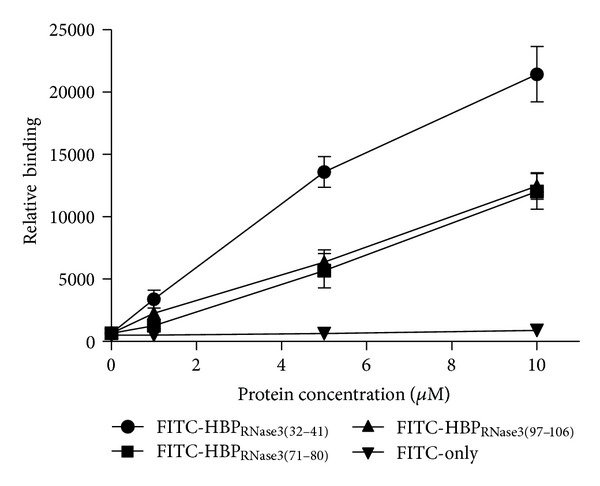
Binding activity of FITC-HBP_RNase3_ peptides to Beas-2B cells. Cellular binding activity of HBPs derived from hRNase3 including FITC-HBP_RNase3(32–41)_, FITC-HBP_RNase3(71–80)_, and FITC-HBP_RNase3(97–106)_ were assayed using cELISA. The level of bound FITC signal was set as negative control. Data represented the means of triplicate incubations. Error bars showed standard deviations (SD) among triplicate experiments.

FITC-labeled HBP_RNase3(32–41)_ (NYRWRCKNQNK), HBP_RNase3(71–80)_ (NNCHRSRFRV) and HBP_RNase3(97–106)_ (TYADRPGRRF), peptides containing, respectively, HBR1 (^34^RWRCK^38^), HBR2 (^73^RSRFR^77^) and HBR3 (^101^RPGRR^105^) sequences were synthesized to investigate the cellular binding activity by cELISA.  Figure 7 indicated that the binding activity of FITC-HBP_RNase3(32–41)_, FITC-HBP_RNase3(71–80)_, and HBP_RNase3(97–106)_ to Beas-2B cells increased with elevated peptide concentration ranging from 1 *μ*M to 10 *μ*M in a dose-dependent manner, the binding activity of FITC-HBP_RNase3(32–41)_ was 2 times stronger than that of FITC-HBP_RNase3(71–80)_ and HBP_RNase3(97–106)_. Since HBR1 was a stronger heparin binding site than HBR2 and HBR3, its multiple functions were further investigated. 

### 3.7. Cellular Binding Activity of hRNase3 and HBP_RNase3(32–41)_

**Figure 8:**
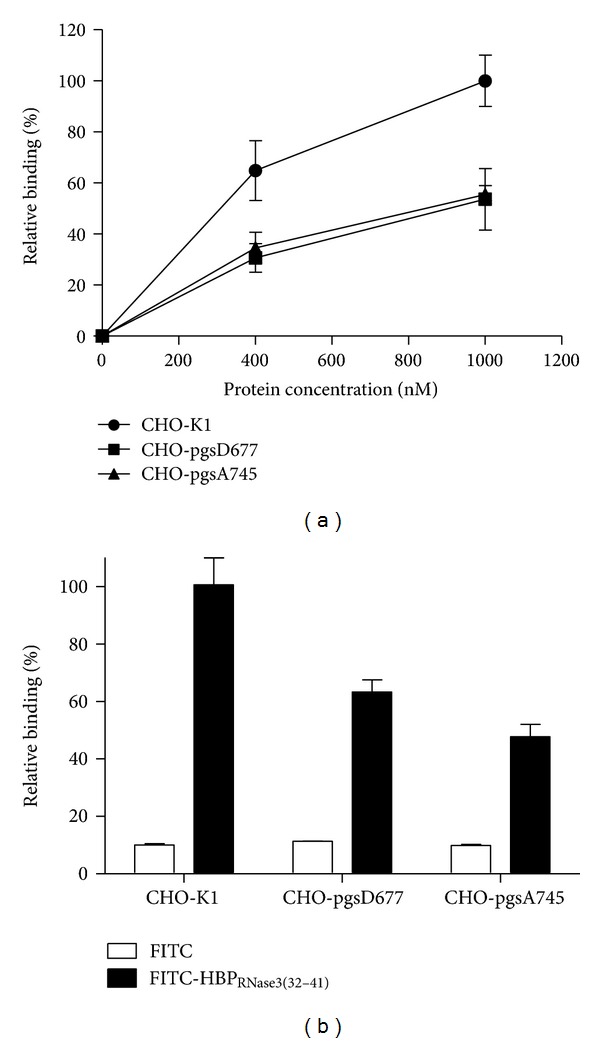
Binding activity of hRNase3 and FITC-HBP_RNase3(32–41)_ to wild-type and mutant CHO cell lines. Cellular binding activity of hRNase3 (a) and FITC-HBP_RNase3(32–41)_ (b) to CHO-K1, CHO-pgsD677, and CHO-pgsA745 cells were assayed using cELISA. The amount of 1000 nM hRNase3 and 5 *μ*M FITC-HBP_RNase3(32–41)_ bound to wild-type CHO-K1cell was set as 100% binding, respectively. The data represented the mean value of triple independent experiments and the error bar was shown as standard deviation (SD).

A series of wild-type and mutant CHO cell lines with specific defect in HS or GAG biosynthesis were employed to study the binding target ofhRNase3 and HBP_RNase3(32–41)_ on plasma membrane.  Figure 8(a) showed that 1000 nM hRNase3 binding to CHO-pgsD677 and CHO-pgsA745 cells was 50% lower than that of wild-type CHO-K1 cells. Similarly, 5 *μ*M FITC-HBP_RNase3(32–41)_ binding to CHO-pgsD677 and CHO-pgsA745 cells significantly decreased, respectively, 40% and 50% as compared to that of wild-type CHO-K1cells (Figure 8(b)), and FITC-only was used a negative control and its relative binding intensities to each cell line as only 10%. While lacking of HS or GAGs on cell surface, relative hRNase3 and HBP_RNase3(32–41)_ binding intensities to both CHO-pgsD677 and CHO-pgsA745 cells dropped about 50%. These results indicated that the presence of HS on the cell surface was most essential for molecular interaction with hRNase3 and HBP_RNase3(32–41)_. In addition, cell surface components, possibly lipid moiety on A745 cells, might be involved in residual hRNase3 and HBP_RNase3(32–41)_ binding activities.

### 3.8. Identification of Specific Lipids Interacting with RNase3 and HBP_RNase3(32–41)_

**Figure 9:**
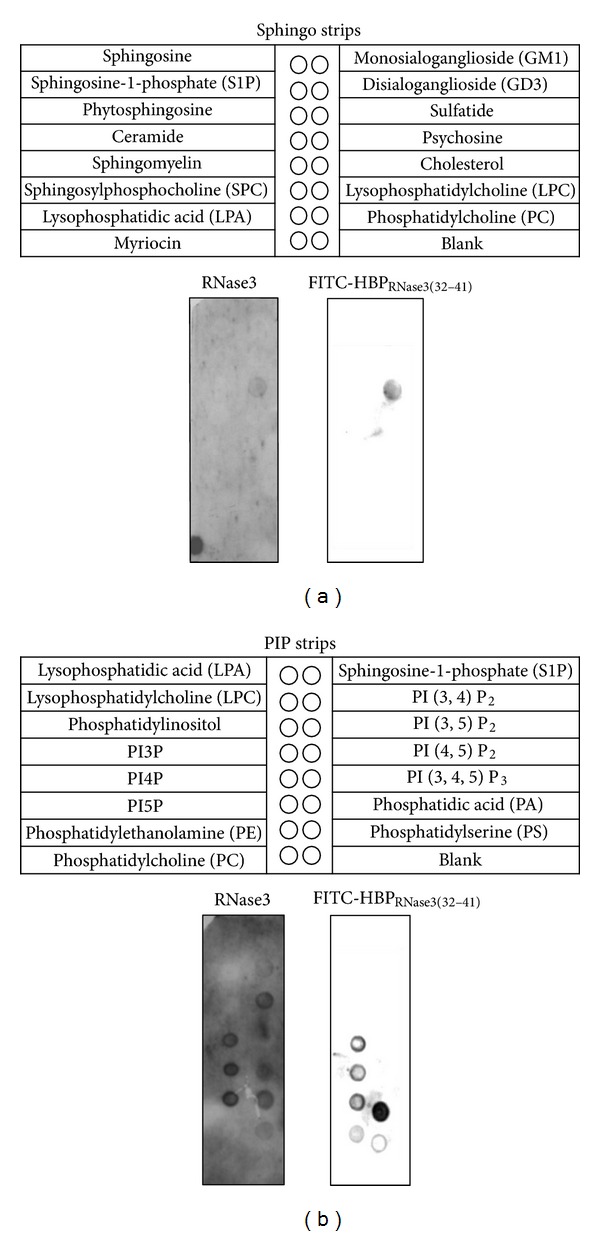
Interaction of hRNase3 and FITC-HBP_RNase3(32–41)_ with membrane lipid fractions. Interaction of hRNase3 and FITC-HBP_RNase3(32–41)_ on lipid overlay blot of SpingoStrips membranes (a) and PIP Strips membranes (b). Membranes were incubated with 1 *μ*g/mL hRNase3 or 0.5 *μ*g/mL FITC-HBP_RNase3(32–41)_ at 37°C for 2 h, separately. The immunoreactive blot of hRNase3 treatment was visualized by enhanced chemiluminescence, and the FITC-HBP_RNase3(32–41)_ treatment was using 488 nm laser with standard 530 nm ± 30 nm bandpass emission filter.

hRNase3 has been associated with lipid membrane destabilization [45]. To investigate whether any other membrane-associated negatively charged moieties other than HS was involved in hRNase3 and HBP_RNase3(32–41)_ binding, overlay blots (SphingoStrips and PIPStrips) containing 100 pmoles of 26 different biologically active sphingolipids and glycerophospholipids were examined [46]. Using SphingoStrips membrane, a specific interaction between sulfatide, a sulfated glycosphingolipid expressed on the cell surface [47], and refold hRNase3 (Figure 9(a), left panel) as well as FITC- HBP_RNase3(32–41)_ (Figure 9(a), right panel) was observed. For the PIP Strips membrane, hRNase3 interacted with PI3P, PI4P, PI5P, PI (3,4) P_2_, PI (3,5) P_2_, PI (3,4,5) P_3_, PA, and PS (Figure 9(b), left panel). FITC- HBP_RNase3(32–41)_ interacted with PI3P, PI4P, PI5P, PE, PA, and PS (Figure 9(b), right panel). These results indicated that FITC-HBP_RNase3(32–41)_ possessed quite similar lipid binding pattern to hRNase3, and the HBP_RNase3(32–41)_ involved in hRNase3 binding to plasma membrane not only through HS binding but also through direct molecular interaction with phospholipids. Our data suggested that hRNase3 interacted with more lipids than HBP_RNase3(32–41)_, presumably due to more diverse lipid binding regions on hRNase3. Besides, variation in the exposed level and conformation of ^32^NYRWRCKNQN^41^ sequence between refold hRNase3 and HBP_RNase3(32–41)_ might also account for the difference in lipid recognition.

### 3.9. Cytotoxicity of hRNase3 and HBP_RNase3(32–41)_ to Beas-2B Cells

**Figure 10:**
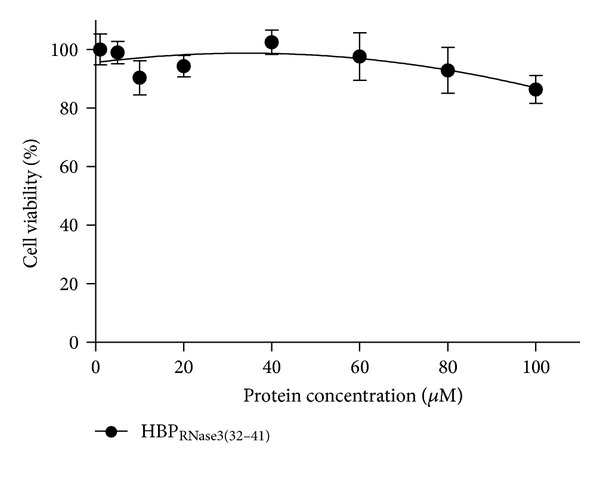
Cell viability of Beas-2B cells upon treatment with HBP_RNase3(32–41)_. Cytotoxicity of HBP_RNase3(32–41)_ to Beas-2B cell was assessed by MTT assay. Beas-2B cells were treated with various concentrations of HBP_RNase3(32–41)_. The amount of cell viability of PBS treated Beas-2B cells was set as 100%.

Cytotoxicity of hRNase3 towards Beas-2B cells has been reported in previous study [27]. To examine the cytotoxic effect of HBP_RNase3(32–41)_, Beas-2B cells were incubated with 0, 5, 10, 20, 40, 60, 80, and 100 *μ*M HBP_RNase3(32–41)_ in serum-free medium at 37°C for 24 h and cell viability was measured by MTT assay.  Figure 10 revealed that no cytotoxicity of HBP_RNase3(32–41)_ was detected, even if high concentration of 100 *μ*M was applied. Thus, although HBP_RNase3(32–41)_ conserved key heparin binding activity of hRNase3, it possessed no cytotoxic effect to mammal cell. 

## 4. Discussion

hRNaseA superfamily members share diverse protein identities to hRNase3 even though they contain conserved 3-dimensional structures and enzymatic functions. In addition to RNase activity, a variety of biological features including immune-regulatory, cytotoxic, antimicrobial, antitumor, and heparin/HS binding activities have been reported. In terms of RNase activities, hRNases1-to-8 have obvious catalytic activities; however, such function is unidentified in hRNases9-to-13 and remains to be elucidated. As for cytotoxicity, hRNases1-to-5 are harmful to various cells or organisms, but hRNases6-to-13 are indistinct. In terms of lipid binding activity, hRNase2, hRNase3, and hRNase7 have been reported to interact with lipids [30–32], while that of the other hRNaseA family members are not well studied. As for antimicrobial activity, hRNase2, hRNase3, hRNase5, hRNase7, and hRNase8 are harmful to microorganisms (Table 1). In addition, hRNase2 and hRNase3 have been elucidated to mediate immune responses [31, 32]. Taken together, comparison of hRNaseA family members indicates that only hRNase2 has similar sequences and functions to hRNase3 (Table 1).

Structural analysis reveals that all hRNaseA family members share very similar secondary structures in three putative HBRs (Table 2). Since hRNase7 shares high primary sequence identity to hRNase6 and hRNase8, 58% and 75%, respectively, its structure was used as a template for structure simulation of hRNase6 and hRNase8 which have no resolved 3D structures yet. The HBR1 and HBR3 in all hRNaseA family members are present, respectively, in loop and *β*-strand conformation. The secondary structure for HBR2 of hRNase4 is *β*-strand, and that of the others is loop conformation. Hence, these results suggested that sequence composition, rather than secondary structure contents of each HBR-like segment in hRNaseA family members, is crucial for differential heparin binding activities. Among all hRNaseA superfamily members, hRNase2, hRNase3, and hRNase7 possess conventional heparin binding motifs in several HBRs. However, only hRNase2, hRNase3, and hRNase5 have been reported to demonstrate heparin binding activities [48, 49]. Here, three unique functional peptides encoded HBRs have been predicted in hRNase3 by ReMUS and demonstrated heparin binding properties at both molecular and cellular levels, and the correspondent HBR1 of hRNase2 has been identified with heparin binding features too. Interestingly, in the primary sequence hRNase5 positively charged residue-rich regions, that is, ^31^RRR^33^ and R^70^ have been reported to involve heparin binding by site-directed mutagenesis [49], and they are located pretty close to putative HBR1 and HBR2 of hRNase5 in this study. Finally, hRNase7 has been reported to be purified through a heparin affinity column despite unclear heparin binding mechanism, indicating that hRNase7 also possesses heparin binding potency. This finding will contribute to further understanding of protein-ligand interaction in hRNaseA members.

hRNase3 is a ribonuclease and its antimicrobial function has been shown to be dependent on its enzymatic activity [5, 19, 25]. Our study has identified three HBRs including ^34^RWRCK^38^, ^73^RSRFR^77^, and ^101^RPGRRR^105^, respectively, located on loop3, loop5, and strand **β**4 of hRNase3 to interact with heparin. Key roles including heparin binding, cytotoxic, endocytic, and lipid binding activities were contributed by these major HBRs and have been demonstrated. hRNase3 can also modulate Beas-2B cells to release TNF-*α* leading to apoptosis facilitated by first step attachment on cell surface GAGs especially HS [26]. In 2010, Garcia-Mayoral group reported that the major HS binding site on hRNase3 was located on a cavity composed by A^8^-Q^14^ in helix *α*1, Y^33^-R^36^ in loop3, Q^40^-L^44^ in strand *β*1, and H^128^-D^130^ in strand *β*6 [18]. Here HBR1 mutants significantly diminished their RNase activity due to replacement of the crucial catalytic residue Lys^38^ to Ala, in addition, it also decreased heparin binding and subsequent endocytosis by replacing three basic residues, Arg^34^, Arg^36^, and Lys^38^ and aromatic residue Trp^35^ responsible for binding and disrupting microbial membrane [50]. Mutation in HBR3 (^101^RPGRR^105^) significantly decreased the cytotoxicity of hRNase3 to Beas-2B cells, revealing that the three cationic residues Arg^101^, Arg^104^, and Arg^105^in HBR3 might play a crucial role in exterminate Beas-2B cells.

HBP_RNase3(31–41)_ segment was demonstrated to be involved in multiple functions of hRNase3 (Table 3). Alignment with primate RNase3 sequences has revealed that the sequence of HBP_RNase3(32–41)_ is conserved only in RNaseA family members of higher primates such as *G. Gorilla* and *P. troglodytes*, the closest living relatives of human. Interestingly, higher primates and the closest living relatives of humans,* P*.* troglodytes *and* G*.* Gorilla* [51] showed 100% sequence identity in corresponding regions to the HBP_RNase3(32–41)_ motif of *H*.* sapiens *RNase3; while *M*.* fascicularis* and *M*.* nemestrina* showed 80% sequence identity with HBP_RNase3(32–41)_ motif of *H*.* sapiens *RNase3 but 100% identity with *H*.* sapiens *RNase2. In addition, the motif sequence of *P*.* pygmaeus *was 70% and 90% identical to that of hRNase3 and hRNase2, respectively (Table 4). Our results strongly supported the notion that hRNase3 was generated from an RNase2/RNase3 precursor gene about 30 million years ago, at an evolutionary rate among the highest primate genes [52].

To date, 13 members of the RNaseA superfamily have been identified in humans; however, the functions of newly identified human hRNases9–13 remain unclear [53]. Blast *analysis *of HBP_RNase3(32–41)_ motif among hRNase3 and other hRNaseA members was shown in Table 5, in which only hRNase2 and hRNase8 showed, respectively, 80% and 50% sequence identity, while the others showed lower than 50% identity with the HBP_RNase3(32–41)_ of hRNase3. In summary, HBP_RNase3_ motif was not a conserved motif among hRNaseA superfamily, but a specific motif being present only in higher primates.

Herein we reported that HBP_RNase3(32–41)_ accounted for major cellular binding activity than HBP_RNase3(71–80)_ and HBP_RNase3(97–106)_. Interestingly, although binding of HBP_RNase3(32–41)_ was severely impaired in CHO-pgsD677 cells which had no HS but expressed 3-fold more CS than wild-type CHO-K1 cells, residual cellular binding activities of HBP_RNase3(32–41)_ to CHO-pgsD677 and CHO-pgsA745 cells strongly implied that HBP_RNase3(32–41)_ possessed certain interaction to cell surface CS. In addition, both hRNase3 and HBP_RNase3(32–41)_ showed quite similar binding activities to membrane lipids. 

## 5. Conclusion

In this study, we identify three functionally important HBRs in hRNase3, including ^34^RWRCK^38^ (HBR1), ^75^RSRFR^79^ (HBR2), and ^101^RPGRR^105^ (HBR3). HBR1 (^34^RWRCK^38^) in hRNase3 is required for RNase activity and serves as a major heparin binding site. HBR2 (^73^RSRFR^77^) contributes to both cell binding and endocytic activities, and HBR3 (^101^RPGRR^105^) plays an important role in cytotoxicity. Moreover, a noncytotoxic HBR1-derived peptide prefers to interact with negatively charged molecules including heparan sulfate, chondroitin sulfate and lipids present on cell surface. Understanding of the roles of key functional residues of hRNase3 in ribonucleolytic, heparin binding, cellular binding, endocytic, cytotoxic, and lipid binding activities provides informative correlation among sequence, structure, and functional features of hRNaseA family members. This finding will contribute to further investigation of molecular mechanisms and multiple functions of hRNaseA family in general.

## Supplementary Material

Supplementary Figure 1: showed that mutant hRNase3 constructs containing alanine replacement in HBRs were generated by site-directed mutagenesis. Replacement with alanine stretch in HBR1, HBR2 and HBR3 were illustrated in black, red, and green boxes, respectively.

## Figures and Tables

**Table 1:** Specific functions of hRNaseA superfamily.

Function	RNase							
	1	2	3	4	5	6	7	8
Protein identity	30%	67%	100%	28%	32%	43%	39%	39%
RNase activity	O	O	O	O	O	O	O	O
Cytotoxicity	O	O	O	O	O	ND	ND	ND
Heparin binding motif	X	O	O	X	O	O	O	X
Lipid binding activity	ND	O	O	ND	ND	ND	O	ND
Antimicrobial activity	ND	O	O	ND	O	ND	O	O
Inflammatory mediators	ND	O	O	ND	ND	ND	ND	ND
Reference	[29]	[30]	[31, 32]	[33, 34]	[35, 36]	[37]	[38, 39]	[40, 41]

Note: O, X, and ND, respectively, represent active, inactive, and not determined.

**Table 2:** Secondary structures of correspondent HBRs in hRNaseA members.

	HBR1	HBR2	HBR3	PDB number
hRNase1	Loop	Loop	*β*-strand	2K11
hRNase2	Loop	Loop	*β*-strand	2C01
hRNase3	Loop	Loop	*β*-strand	2KB5
hRNase4	Loop	*β*-strand	*β*-strand	1RNF
hRNase5	Loop	Loop	*β*-strand	1H53
hRNase6	Loop	Loop	*β*-strand	2HKY*
hRNase7	Loop	Loop	*β*-strand	2HKY
hRNase8	Loop	Loop	*β*-strand	2HKY*

The structures of hRNase1, 2, 3, 4, 5, and 7 are collected from protein data bank (PDB), and those of RNase6, 8 were simulated by database (PS)^2^—Protein Structure Prediction Server (http://ps2.life.nctu.edu.tw/). *Denotes simulated structures using hRNase7 as template.

**Table 3:** Correlation of HBR motifs and characteristic functions of hRNase3.

Location	Function					
	RNase	Cytotoxicity	Cell binding activity	HS binding activity	Endocytic activity	Lipid binding activity
RNase3	O	O	O	O	O	O
HBR1-mt RNase3	No activity	Decrease 50%	Increase 50%	Decrease 40%	No activity
HBR2-mt RNase3	Similar	Similar	Decrease 20%	Similar	Decrease 50%
HBR3-mt RNase3	Decrease 30%	No activity	Decrease 20%	Similar	Similar
HBP_RNase3(32–41)_	X	X	O	O	ND	O

Note: O, X, and ND, respectively, represent active, inactive, and not determined.

**Table 4:** Comparison of HBP_RNase3(32–41)_ motif among hRNase3 and other primate RNase3s and RNase2s.

Protein	Organism	Protein identity	HBP_RNase3(32–41)_ identity	Sequence
RNase3	*Homo sapiens *	100%	100%	N YRWRCKNQN
	*Pan troglodytes *	97%	100%	NYRWRCKNQN
	*Gorilla gorilla *	97%	100%	NYRWRCKNQN
	*Macaca fascicularis *	88%	80%	NYQRRCKNQN
	*Macaca nemestrina *	88%	80%	NYQRRCKNQN
	*Pongo pygmaeus *	88%	70%	NYQRRCKDQN

RNase2	*Homo sapiens *	67%	80%	NYQRQCKNQN
	*Pan troglodytes *	67%	80%	NYQRQCKNQN
	*Gorilla gorilla *	69%	80%	NYQRQCKNQN
	*Macaca fascicularis *	67%	80%	NYQRQCKNQN
	*Macaca nemestrina *	66%	80%	NYQRQCKNQN
	*Pongo pygmaeus *	68%	70%	NFQRRCKNQN

Sequence identity was calculated employing National Center for Biotechnology Information Blast (NCBI Blast: http://blast.ncbi.nlm.nih.gov/Blast.cgi).

**Table 5:** Comparison of HBP_RNase3(32–41)_ motifs among human RNaseA superfamily members.

Name	Accession number	Protein identity	HBP_RNase3(32–41)_ identity	Sequence
RNase1	*P07998*	30%	40%	M TQGRCKPVN
RNase2	*P10153*	67%	80%	NYQRRCKNQN
RNase3	*P12724*	100%	100%	NYRWRCKNQN
RNase4	*P34096*	28%	30%	MTLYHCKRFN
RNase5	*P03950*	32%	30%	LTSP - CKDIN
RNase6	*Q93061*	43%	20%	KYFGRSLELY
RNase7	*Q9H1E1*	39%	40%	KHTKRCKDLN
RNase8	*Q8TDE3*	39%	50%	KYTERCKDLN
RNase9	*P60153*	23%	20%	YYKHRWVAEH
RNase10	*Q5GAN6*	26%	10%	EPSQSCIAQY
RNase11	*Q5GAN5*	30%	30%	EANGSCKWSN
RNase12	*Q5GAN4*	23%	20%	EPDHTCKKEH
RNase13	*Q5GAN3*	25%	10%	MQNSDCPKIH

Sequence identity was calculated employing National Center for Biotechnology Information Blast (NCBI Blast: http://blast.ncbi.nlm.nih.gov/Blast.cgi).
