# Supplementary figures and images for: In Silico Prediction and In Vitro Characterization of Multifunctional Human RNase3
Source: Biomed Res Int. 2013 Jan 17;2013:170398. doi: 10.1155/2013/170398 (PMC3581242; doi:10.1155/2013/170398)

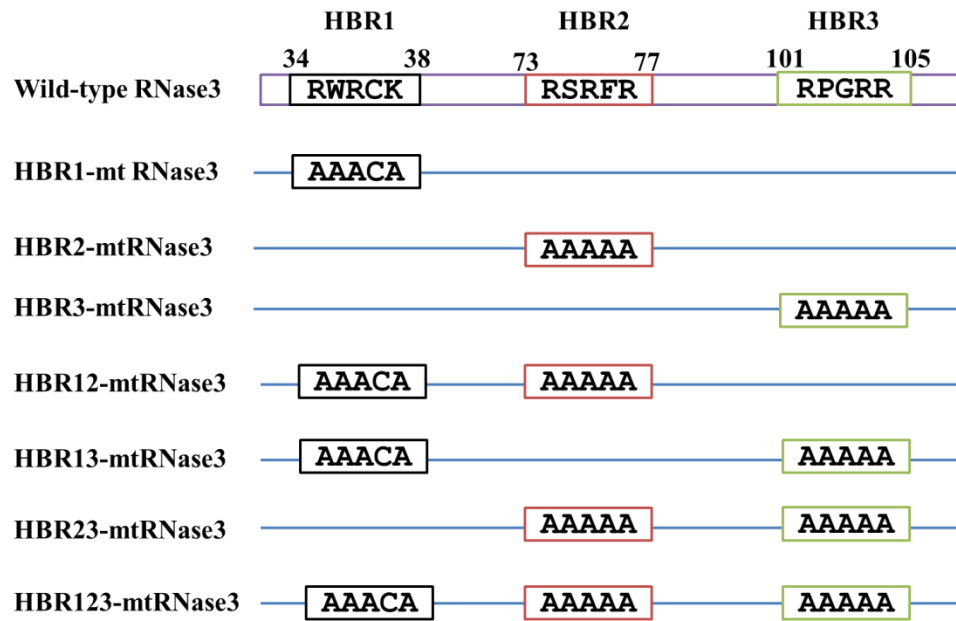

Supplement: Supplementary file 1 — Supplementary Figure 1: showed that mutant hRNase3 constructs containing alanine replacement in HBRs were generated by site-directed mutagenesis. Replacement with alanine stretch in HBR1, HBR2 and HBR3 were illustrated in black, red, and green boxes, respectively. [file 170398.f1.pdf]
